# Associations between intelligence, affective temperament, and parental bonding with educational level

**DOI:** 10.3389/fgwh.2025.1648474

**Published:** 2025-09-12

**Authors:** Hirofumi Hirakawa, Takeshi Terao, Kentaro Kohno, Akari Sakai, Nobuko Kawano

**Affiliations:** 1 Department of Neuropsychiatry, Oita University Faculty of Medicine, Yufu, Oita, Japan; 2 Oita Occupational Health Management Center, Nishinihon Occupational Health Center, Oita, Oita, Japan; 3 Department of Psychology, Oita University Faculty of Welfare and Health Sciences, Oita, Oita, Japan

**Keywords:** educational level, maternal care, intelligence, age, male

## Abstract

**Background:**

Educational level is an important aspect of one's life and is associated with biopsychosocial and economic factors. The present study aimed to investigate the association of educational level with intelligence, affective temperament, and parental bonding.

**Methods:**

This is a cross-sectional study. The dataset included 130 individuals consisting of 20 with high school education level, 51 with college education level, and 59 with university or higher education level. First, demographic data, including intelligence, affective temperament, and parental bonding were compared among the three groups using analysis of variance (ANOVA). Second, multiple regression analysis using stepwise method was performed, with educational levels as dependent variable and significant variables in the ANOVA as independent variables.

**Results:**

Intelligence, maternal care, and male sex were significantly and positively associated with educational level, whereas age was significantly and negatively associated with educational level.

**Conclusion:**

The present findings suggest that maternal care as well as intelligence may be associated with higher educational level. Further prospective studies are required to determine any causal relationship and investigate other factors related to educational level.

## 1 Introduction

Educational level is an important aspect of one's life and is associated with biopsychosocial and economic factors. When high school students determine whether they enter college, university, or not, they may think by themselves and consult their teachers, friends, and parents. In the course of decision-making, their temperament and their relationship with parents may be crucial. Moreover, their intelligence may be one of the most important factors for educational level.

Liu et al. (1) indicated that, in China, when mothers hold dominant positions in their children's educational decisions, they are more likely to adopt a “tiger mom” approach. Recently, maternal dominance in household decision-making has increased due to social development and evolving gender roles. This shift is reflected in the emergence of various family models, such as the dynamics of “Tiger Mom and Cat Dad”, “Wife Dominant and Husband Accompanying”, and “Supermom.” Therefore, exploring the impact of maternal dominance in children's educational decision-making on the development of adolescents' human capital is essential (1).

As for parental bonding, Ling et al. (2) showed that parental antipathy and neglect and mother negative loving (a form of insecure attachment) sequentially mediated the association between maternal control and adulthood antisocial personality disorder features. Geng et al. (3) suggest that it is important to pay attention to medical students (especially women) with low maternal care and low empathy (cognitive empathy in particular), and that these students are more likely to have depressive symptoms during their medical studies and clinical work. In UK, Reyes et al. (4) showed that lower maternal attachment in early life predicted increased adolescent multiple risky behaviors, and that almost a third of the excess risk was attributable to child, maternal and socioeconomic factors, with over a quarter explained by maternal depression.

In USA, Wegemer and Vandell (5) investigated early childhood antecedents of adult political orientation using longitudinal data, and found that mothers' beliefs and parenting behaviors, children's temperament, and attachment security during early childhood were related to political ideology and party affiliation at age 26. Young children's fearful temperament and anxious attachment security, as well as mothers' authoritarian parenting beliefs in early childhood, predicted conservative political orientations at age 26. Children's abilities to focus attention and avoidant attachment security predicted liberal orientations (5).

These findings are not directly associated with educational level, but possibly indirectly associated with the level via decision-making, which may be affected by intelligence, affective temperament, and parental bonding. In the present study, we hypothesized that higher educational level may be associated with higher intelligence, hyperthymic temperament, and more maternal care. The aim of this study was to investigate this hypothesis.

## 2 Materials and methods

### 2.1 Subjects

We used data from a previous study (6) which was a prospective longitudinal study on psychotherapy in apparently healthy participants. In the previous study, the inclusion criterion was individuals aged ≥20 years who provided written informed consent. Participants were recruited via electronic bulletin boards, physical bulletin boards, and flyers. Individuals with serious psychiatric disorders, as determined by the mini-international neuropsychiatric interview (M.I.N.I.), were excluded. The Institutional Review Board of Oita University Faculty of Medicine approved the trial (number B16-023). All participants provided written informed consent.

Although the previous study (6) had 137 subjects, our previous opt-out study (7) excluded 7 subjects and created the dataset of 130 subjects because of missing data. In this study, we used the dataset of 130 subjects, which included 108 females and 22 males, with a mean age of 49.3 years (SD = 12.1). This opt-out study was approved by the ethics committee of the Oita University Faculty of Medicine on June 12, 2023 (approval number 2536).

It should be noted that the dataset was originally obtained from a prospective longitudinal study (6), but that our current analysis was a cross-sectional study.

### 2.2 Educational level

Educational level was categorized into HE (high school education level), CE (college education level), and UE (university or higher education level). There were 20 individuals (female 19: male 1) in category 1, 51 (49: 2) in category 2, and 59 (40: 19) in category 3. The sex distribution along with educational level was significantly deviated (*χ*^2^ = 18.0, *p* < 0.001). It should be noted that category UE included three medical students.

### 2.3 Intelligence

The National Adult Reading Test (NART) (8) is widely used as a measure of premorbid IQ of the English-speaking patients with dementia. There is a Japanese version of the NART (JART), using 50 Japanese irregular words, all of which are Kanji (ideographic script) compound words. The JART has been translated into Japanese, and the reliability and validity of the Japanese version have been established (9).

### 2.4 Affective temperament

The Japanese version of the Temperament Evaluation of Memphis, Pisa, Paris, and the San Diego-auto questionnaire (TEMPS-A), a 110-item true–false questionnaire that assesses five temperament dimensions: depressive, cyclothymic, hyperthymic, irritable, and anxious (10). TEMPS-A has been translated into Japanese, and the reliability and validity of the Japanese version have been established (11).

### 2.5 Bonding style

Parental Bonding Instrument (PBI) is a retrospective self-assessment measure of parental rearing attitudes experienced in childhood (12). The PBI consists of two subscales of “care” and “overprotection.” A high score for “care” indicates more appropriate rearing (less rejection and indifference), whereas a high score for “overprotection” indicates inappropriate overprotection (less encouragement of self-reliance). The PBI consists of 25 items (12 items for care and 13 items for overprotection), and each item is assessed on a 4-point Likert scale. The PBI has been translated into Japanese, and the reliability and validity of the Japanese version have been established (13, 14).

### 2.6 Data analysis

First, analysis of variance (ANOVA) was used to compare the demographic data (age, parental bonding style, affective temperament, intelligence) across educational level (three category groups). Second, multiple regression analyses using stepwise method were conducted with educational level as the dependent variable and sex and ANOVA results (*P* < 0.05) as independent variables.

## 3 Results

Table 1 shows the data of age, parental bonding style, affective temperament, intelligence across the three groups along with the results of ANOVA. Age, paternal overprotection, maternal care, irritable temperament, and intelligence were significantly different across the three groups.

**Table 1:** Demographic data, including age, parental bonding, affective temperament, and intelligence (JARTIQ) across educational level and results of ANOVA.

Category		*N*	mean	SD	min	max	ANOVA
Age	HE	20	57.40	9.121	42	77	
	CE	51	49.29	10.734	27	77	
	UE	59	46.44	13.033	25	77	F = 6.63
	total	130	49.25	12.132	25	77	*p* = 0.002**
Paternal care	HE	20	20.90	8.410	3	35	
	CE	48	19.98	9.604	0	36	
	UE	56	23.16	8.132	1	36	F = 1.77
	total	124	21.56	8.829	0	36	*p* = 0.18
Paternal overprotection	HE	20	12.90	7.018	0	25	
	CE	49	12.88	7.216	0	33	
	UE	55	9.42	6.115	0	26	F = 4.08
	total	124	11.35	6.880	0	33	*p* = 0.019*
Maternal care	HE	18	20.17	8.248	5	34	
	CE	49	23.33	6.694	7	35	
	UE	57	26.37	7.442	6	36	F = 5.64
	total	124	24.27	7.547	5	36	*p* = 0.005**
Maternal overprotection	HE	20	10.85	6.499	1	21	
	CE	50	12.88	7.122	0	28	
	UE	57	10.51	7.944	0	36	F = 1.45
	total	127	11.50	7.444	0	36	*p* = 0.24
Depressive temperament	HE	20	9.30	4.105	1	17	
	CE	51	9.04	3.493	2	15	
	UE	58	7.91	3.268	0	16	F = 1.92
	total	129	8.57	3.520	0	17	*p* = 0.16
Cyclothymic temperament	HE	19	3.68	3.449	0	15	
	CE	51	4.39	3.873	0	14	
	UE	58	5.66	4.625	0	18	F = 2.12
	total	128	4.86	4.216	0	18	*p* = 0.13
Hyperthymic temperament	HE	20	4.20	2.895	0	9	
	CE	50	5.02	3.634	0	18	
	UE	58	5.48	3.283	0	15	F = 1.10
	total	128	5.10	3.374	0	18	*p* = 0.34
Irritable temperament	HE	19	1.21	1.512	0	6	
	CE	51	3.45	3.618	0	12	
	UE	58	3.07	2.979	0	11	F = 3.70
	total	128	2.95	3.166	0	12	*p* = 0.028*
Anxious temperament	HE	20	7.05	4.718	1	19	
	CE	51	7.18	6.085	0	24	
	UE	57	6.25	4.801	0	17	F = 0.45
	total	128	6.74	5.317	0	24	*p* = 0.64
JARTIQ	HE	20	105.15	9.382	90	119	
	CE	51	107.08	7.177	92	119	
	UE	59	113.37	5.258	99	124	F = 16.93
	total	130	109.64	7.580	90	124	*P* < 0.001***

Category: 1 (high school educational level: HE), 2 (college educational level: CE), 3 (university or higher educational level: UE).

* *p* < 0.05.

** *p* < 0.01.

*** *p* < 0.001.

Table 2 shows the results of multiple regression analysis, where intelligence, maternal care, and male sex were significantly and positively associated with educational level, whereas age was significantly and negatively associated with educational level. On the other hand, paternal overprotection and irritable temperament were excluded from the model.

**Table 2:** Final model by multiple regression analysis for educational level using stepwise method.

Variables	*β*	*t*	*p*
JARTIQ	0.391	5.154	0.000
Age	−0.257	−3.385	0.001
Sex (1 = female, 2 = male)	0.267	3.571	0.001
Maternal care	0.155	2.021	0.046

Adjusted *R*^2^ = 0.353, *F* = 17.1, *p* = 0.001.

JARTIQ, Japanese adult reading test intelligence quotient.

*β*, standardized partial regression coefficient which shows whether the association is positive. negative, strong or weak.

## 4 Discussion

The major findings of this study can be summarized as follows: (1) higher intelligence and more maternal care were significantly associated with higher educational level. (2) male sex and younger age were significantly associated with higher education level. To our knowledge, this is the first study investigating associations between intelligence, affective temperament, and parental bonding concurrently with educational level.

With regard to our hypothesis that higher educational level may be associated with higher intelligence, hyperthymic temperament, and more maternal care, the involvement of higher intelligence and more maternal care with higher education level is in agreement with the present results. Moreover, the importance of maternal care is in agreement with the previous reports (2–5). However, unexpectedly, hyperthymic temperament was not associated with educational level. Although the reason is uncertain, hyperthymic temperament may be independent from any educational level. As shown in Table 1, however, the scores of hyperthymic temperament and cyclothymic temperament increased along with the increase of educational level, although they were not significant. Possibly, this may suggest the modest involvement of common factor such as ambition between hyperthymic and cyclothymic temperament with educational level. Moreover, the “modest” involvement may be derived from the fact that majority of the subjects were females (108 females and 22 males). This is probably because hyperthymic male individuals can express themselves as they want, whereas hyperthymic female individuals can express themselves within the limited social culture where male dominance is still prevalent at least in Japan. Interestingly, this is contrast to “tiger mom” in China (1).

As for the involvement of male sex and younger age with higher education, in old Japan, male dominance was prevalent and female individuals were less likely to enter into higher education level. Since the present subjects had mainly females, the results may reflect such tendency in older individuals. Also, the fact that three medical students were included in the subjects may contribute to the negative association between age and educational level and the positive association between intelligence and educational level. In order to investigate this possibility, we excluded these medical students, but the results were almost unchanged (data not shown).

This study had some limitations. First, this was a cross-sectional study, and causal relationships could not be determined. Second, the sample size was relatively precluded to establish a definite conclusion. Third, JARTIQ could not measure total intelligence. Forth, the sample was heavily skewed towards females (108 females vs. 22 males), which limits generalizability. Because of small sample of males, however, we could not perform additional sex-stratified analysis. Fifth, recruitment through bulletin boards and flyers might have introduced selection bias. Sixth, the negative association between age and educational level could be due to cohort effects in access to education. Seventh, further studies are required to clarify why male sex and younger age were significantly associated with higher education level and conversely why female sex and older age were not associated with higher educational level. Finally, this study focused on only psychological aspects, and biological, social, economic, and cultural aspects should be considered as well. Particularly, important potential confounders such as socioeconomic status, parental occupation, and geographic location were not included. These factors could influence both maternal care and educational attainment.

In conclusion, the present findings suggest that maternal care as well as intelligence may be associated with higher educational level. Further prospective studies are required to determine any causal relationship and investigate other factors related to educational level stratified by sex.

## Data Availability

The data analyzed in this study is subject to the following licenses/restrictions. The data may be available upon request to the corresponding author. Requests to access these datasets should be directed to Takeshi Terao, terao@oita-u.ac.jp.

## References

[1] Liu C Ren F Yang L Fan W Huang X. Cognitive or non-cognitive? The effect of maternal dominance on adolescent human capital: evidence from adolescents’ educational decisions. Econ Hum Biol. (2025) 56:101463. 10.1016/j.ehb.2024.101463 39765046

[2] Ling H Meng F Yan Y Feng H Zhang J Zhang L Why is maternal control harmful? The relation between maternal control, insecure attachment and antisocial personality disorder features in Chinese college students: a sequential mediation model. Int J Environ Res Public Health. (2022) 19(17):10900. 10.3390/ijerph191710900 36078615 PMC9518312

[3] Geng Y Fei W Tang Z Wang S Yu J Zhang M Parental care and depressive symptoms among Chinese medical students: roles of empathy and gender. BMC Med Educ. (2022) 22(1):451. 10.1186/s12909-022-03524-2 35689260 PMC9188078

[4] Reyes BD Hargreaves DS Creese H. Early-life maternal attachment and risky health behaviours in adolescence: findings from the United Kingdom millennium cohort study. BMC Public Health. (2021) 21(1):2039. 10.1186/s12889-021-12141-5 34749702 PMC8577004

[5] Wegemer CM Vandell DL. Parenting, temperament, and attachment security as antecedents of political orientation: longitudinal evidence from early childhood to age 26. Dev Psychol. (2020) 56(7):1360–71. 10.1037/dev0000965 32378919 PMC8274710

[6] Sakai A Terao T Kawano N Akase M Hatano K Shirahama M Existential and mindfulness-based intervention to increase self-compassion in apparently healthy subjects (the EXMIND study): a randomized controlled trial. Front Psychiatry. (2019) 10: 538. 10.3389/fpsyt.2019.00538 31427998 PMC6687764

[7] Eto M Terao T Satoh M Kawano N Sakai A Akase M The association of self-compassion with depressive temperament and Reading habit. Acad Ment Health Well-Being. (2024) 1. 10.20935/MHealthWellB6176

[8] Nelson H**.** The National Adult Reading Test (NART) Test Manual. Windsor, UK: NFER-Nelson (1978).

[9] Matsuoka K Uno M Kasai K Koyama K Kim Y. Estimation of premorbid IQ in individuals with Alzheimer’s disease using Japanese ideographic script (Kanji) compound words: Japanese version of national adult reading test. Psychiatry Clin Neurosci. (2006) 60:332–9. 10.1111/j.1440-1819.2006.01510.x 16732750

[10] Akiskal HS Akiskal KK Haykal RF Manning JS Connor PD. TEMPS-A: progress towards validation of a self-rated clinical version of the temperament evaluation of the Memphis, Pisa, Paris, and San Diego autoquestionnaire. J Affect Disord. (2005) 85:3–16. 10.1016/j.jad.2004.12.001 15780671

[11] Matsumoto S Akiyama T Tsuda H Miyake Y Kawamura Y Noda T Reliability and validity of TEMPS-A in a Japanese non-clinical population: application to unipolar and bipolar depressives. J Affect Disord. (2005) 85:85–92. 10.1016/j.jad.2003.10.001 15780679

[12] Parker G Tupling H Brown LB. A parental bonding instrument. Br J Med Psychol. (1979) 52:1–10. 10.1111/j.2044-8341.1979.tb02487.x

[13] Ogawa M. A study of the reliability and validity of the Japanese version of the PBI (parental bonding instrument). Jpn J Psychiatri Treat. (1991) 6:1193–201.

[14] Kitamura T Suzuki T. A validation study of the parental bonding instrument in a Japanese population. Jpn J Psychiatry Neurol. (1993) 47:29–36. 8411788 10.1111/j.1440-1819.1993.tb02026.x

